# CCp5A Protein from *Toxoplasma gondii* as a Serological Marker of Oocyst-driven Infections in Humans and Domestic Animals

**DOI:** 10.3389/fmicb.2015.01305

**Published:** 2015-11-24

**Authors:** Silas S. Santana, Luiz C. Gebrim, Fernando R. Carvalho, Heber S. Barros, Patrício C. Barros, Ana C. A. M. Pajuaba, Valeria Messina, Alessia Possenti, Simona Cherchi, Edna M. V. Reiche, Italmar T. Navarro, João L. Garcia, Edoardo Pozio, Tiago W. P. Mineo, Furio Spano, José R. Mineo

**Affiliations:** ^1^Laboratory of Immunoparasitology, Institute of Biomedical Sciences, Federal University of UberlândiaUberlândia, Brazil; ^2^Department of Infectious, Parasitic and Immunomediated Diseases, Istituto Superiore di SanitàRome, Italy; ^3^Department of Clinical Medicine, State University of Londrina – University HospitalLondrina, Brazil; ^4^Department of Preventive Veterinary Medicine, State University of LondrinaLondrina, Brazil

**Keywords:** *Toxoplasma gondii*, oocyst-sporozoite antigen, molecular marker of infection, human toxoplasmosis, animal toxoplasmosis, food security

## Abstract

Considering that the current immunoassays are not able to distinguish the infective forms that cause *Toxoplasma gondii* infection, the present study was carried out to evaluate the reactivity of two recombinant proteins (CCp5A and OWP1) from oocyst/sporozoite, in order to differentiate infections occurring by ingestion of oocysts or tissue cysts. The reactivity of the recombinant proteins was assessed against panels of serum samples from animals (chickens, pigs, and mice) that were naturally or experimentally infected by different infective stages of the parasite. Also, we tested sera from humans who have been infected by oocysts during a well-characterized toxoplasmosis outbreak, as well as sera from pregnant women tested IgM^+^/IgG^+^ for *T. gondii*, which source of infection was unknown. Only the sporozoite-specific CCp5A protein was able to differentiate the parasite stage that infected chickens, pigs and mice, with specific reactivity for oocyst-infected animals. Furthermore, the CCp5A showed preferential reactivity for recent infection by oocyst/sporozoite in pigs and mice. In humans, CCp5A showed higher reactivity with serum samples from the outbreak, compared with serum from pregnant women. Altogether, these findings demonstrate the usefulness of the CCp5A protein as a new tool to identify the parasite stage of *T. gondii* infection, allowing its application for diagnosis and epidemiological investigations in animals and humans. The identification of parasite infective stage can help to design effective strategies to minimize severe complications in immunocompromised people and, particularly, in pregnant women to prevent congenital infection.

## Introduction

*Toxoplasma gondii* is an apicomplexan ubiquitary intracellular parasite that can infect all warm-blooded animals, including humans (Tenter et al., 2000; Dubey et al., 2012; Franco et al., 2015). The infection caused by this parasite is usually asymptomatic for immunocompetent individuals, but it causes severe consequences for immunocompromised individuals, like HIV–AIDS patients. Also, when pregnant women acquire primary infection and the parasite crosses the placental barrier, the fetus could be severely affected, which may lead to abortion, neonatal death, or significant postnatal complications (Halonen and Weiss, 2013).

The definitive hosts of this parasite are members of the Felidae family, while the intermediate hosts are countless warm-blooded animals. All three parasite stages are able to infect the hosts: the bradyzoites present in tissue cysts, the sporozoites contained in the oocysts and the tachyzoites (Innes, 2010). The oral route is the most frequent form of postnatal transmission of toxoplasmosis in humans. The infection can occur through ingestion of infective oocysts shed with cat feces, which are present in the environment, on vegetables, water and litter boxes, or by ingestion of raw/undercooked meat (from pork, lamb, chicken, or goat) containing tissue cysts. The prevalence of infections is strictly related to cultural habits, food quality, hygiene, and socio-economic status (Dubey, 1986, 2009; Aramini et al., 1999; Bahia-Oliveira et al., 2003; Innes et al., 2009; Innes, 2010).

The oocyst stage of *T. gondii* is highly resistant to disinfectants or freezing, although heat above 55°C may kill the sporozoites (Hill and Dubey, 2002; Schluter et al., 2014), and contaminated drinking water sources may cause outbreaks of toxoplasmosis worldwide. Thus, oocysts are considered an important source of environmental contamination (Dubey, 2004; Jones and Dubey, 2010). Until a few years ago, waterborne toxoplasmosis had been considered rare, but this assertion can be considered inappropriate nowadays, because there have been several outbreaks in recent years linked to *T. gondii* transmission by water (Dubey et al., 2012). In 1979, a toxoplasmosis outbreak occurred among US military members, who were doing training in Panama. It was attributed to the water contaminated by feces from wild cats as the source of infection, considering that the soldiers were fed only by controlled foods (Sulzer et al., 1986). In 1995, an extensive outbreak in Victoria, Canada, was connected to water infection via oocyst (Bowie et al., 1997; Burnett et al., 1998). Since 1990, oocysts have been implicated as infective stage in toxoplasmosis outbreaks in Brazil. In Santa Isabel do Ivaí, from 426 individuals presenting anti-*Toxoplasma* IgM and IgG, 176 fulfilled the criteria to be defined as cases, and 155 of these patients showed symptoms of acute toxoplasmosis. In this outbreak, the patients were infected by *T. gondii* oocysts excreted by feces from domestic cats, which contaminated an underground reservoir containing unfiltered water (de Moura et al., 2006).

In view of the great medical importance of toxoplasmosis, many methods have been developed in the last decades to improve the accuracy and sensitivity of serological assays. Whole tachyzoite extracts have been used as antigen in various protocols, resulting in significant limitations for standardization of the serological assays. For this reason, selected *T. gondii* recombinant proteins have great potential as immunoreagents (Beghetto et al., 2003; Buffolano et al., 2005; Kotresha and Noordin, 2010; Hill et al., 2011; Santana et al., 2012). The use of these proteins makes possible to design new diagnostic tests based on well-characterized antigens. Additional advantages include the relative low cost, high degree of purity, and the possibility to select specific antigens for a given infective stage of the parasite (Pietkiewicz et al., 2004, 2007; Pfrepper et al., 2005; Kotresha and Noordin, 2010; Hill et al., 2011).

Currently, there is no stage-specific serological assay for toxoplasmosis to estimate the sources of infection worldwide. The lack of this information hampers the accomplishment of procedures to prevent and control *T. gondii* infection, as the evidences of infection via oocyst is exclusively based on epidemiological surveillance studies, which can be inaccurate in many situations. These facts have also restricted the implementation of educational programs to reduce or minimize the risk factors associated with toxoplasmosis (Hill et al., 2011; Munoz-Zanzi et al., 2012).

Recent proteomic studies have shed light on the repertoire of proteins expressed by a so far poorly characterized stage of *T. gondii*, the oocyst/sporozoite (Possenti et al., 2010, 2013; Fritz et al., 2012a,b). Some of the stage-specific components identified by this strategy can be considered as potential targets for the development of novel diagnostic immunoassays able to identify oocyst-driven infections. In this context, the major aim of the present study was to assess the antigenic reactivity of TgOWP1 and TgCCp5A, two polypeptides specifically expressed by *T. gondii* oocysts or sporozoites, respectively. Recombinant forms at these molecules were assayed, in parallel with soluble tachyzoite antigen, against panels of serum samples from the following hosts: chickens (naturally infected by oocysts, experimentally immunized with tachyzoite antigen, or experimentally infected with tissue cysts); pigs (naturally infected or experimentally infected with oocysts or tachyzoites); mice (experimentally infected with cysts or oocysts); and humans (pregnant women positive for anti-*T. gondii* IgM/IgG and individuals infected by oocysts during an outbreak of toxoplasmosis). It was found that the CCp5A recombinant polypeptide was able to efficiently differentiate the parasite stage in infected chickens, pigs and mice, since stage-specific antibodies were detected only in the sera from oocyst-infected animals. In addition, human serum samples from individuals who acquired infection by oocyst ingestion showed higher IgM and IgG antibody levels to CCp5A in comparison with serum samples from pregnant women. Together, these findings indicate that CCp5A is a good molecular marker to differentiate the *T. gondii* infective stages responsible for infection.

## Materials and Methods

### Study Approval

The maintenance and care of experimental animals complied with the National Institutes of Health guidelines for the human use of laboratory animals. The Ethics Committee for Animal Experimentation from Federal University of Uberlândia (CEUA-UFU) approved all procedures. Hens and mice were maintained in individual cages at animal facilities from this institution, and they received food and water ad libitum. Pigs were randomly allocated in separate stables and all procedures involving these animals were approved for the Ethics Committee for Animal Experimentation of State University of Londrina, Paraná, Brazil. Concerning the experiments analyzing human sera, the study was approved by the Ethical Committee Involving Humans from the State University of Londrina (CEP-UEL) and all patients signed the consent term prior they have been enrolled in this investigation.

### Proteins

#### Soluble Toxoplasma Antigen (STAg)

Parasite suspensions from RH strain were adjusted to 1 × 10^8^ tachyzoites/ml and submitted to freeze-thawing and sonication cycles in the presence of protease inhibitors (Mineo et al., 1980; Marcolino et al., 2000). The preparation was centrifuged at 10.000 × *g* for 15 min at 4°C, and the supernatants were collected. Subsequently, the protein content was determined (Bradford, 1976) and aliquots of 100 ml were stored at -20°C until being tested.

#### Recombinant Proteins

Recombinant CCp5A and OWP1 possessing an N-terminal tail of six histidines were produced according to the strategy described previously (Possenti et al., 2010). Proteins were purified by nickel affinity chromatography and their contents quantified (Bradford, 1976). Aliquots of 100 μL were stored at -20°C until being used. The integrity of recombinant proteins and STAg were assessed by electrophoresis on 12% minigels.

### Serum Samples from Animals

#### Chicken Serum Samples

Chicken serum samples were obtained from animals naturally infected by oocyst, experimentally immunized with STAg or experimentally infected with tissue cyst. Serum samples from naturally infected chickens were collected from chickens raised extensively (*n* = 113) in various suburban areas from Uberlândia city, MG, Brazil. Chickens raised extensively that were seropositive to *T. gondii* were considered naturally infected. For serum samples of animals that were experimentally infected or immunized, hens of a commercial strain were used. The hens were derived from chickens immunized against the various pathogens, but they were seronegative to *T. gondii* infection. Serum samples from chickens immunized with STAg were collected after immunization of hens with emulsion composed of STAg and Freund’s adjuvant by muscular route. Primary immunization was performed with 100 μg of STAg in 250 μL of PBS and equal volume of Freund’s complete adjuvant (Sigma Chem, Co., St. Louis, MO, USA). Two boosters were performed at 15-days intervals, with 100 μg of STAg plus Freund’s incomplete adjuvant, as described elsewhere (Ferreira-Júnior et al., 2012). Serum samples from experimentally infected chickens with cysts were obtained after oral infection of hens with 100 cysts of *T. gondii* (VEG strain), as previously described (Kaneto et al., 1997).

#### Pig Serum Samples

Serum samples were obtained from three groups of pigs, i.e., naturally infected by oocyst, experimentally infected with tachyzoites or experimentally infected with oocysts. Samples from naturally infected pigs were collected from 44 castrated male pigs with the same genetic background. These animals were weaned when they were 21-days-old and maintained at Piracaíba farm, Araguari, MG, Brazil. For the serum samples of animals that were experimentally infected, six mixed-breed pigs from 6.5 to 7.5 weeks of age, including females and castrated males, were randomly allocated in separate stables. The animals were acclimatized for 6 days. All pigs were seronegative to *T. gondii* until the day of infection. The pigs were experimentally infected with 1 × 10^6^ tachyzoites of RH strain by intramuscular injection (*n* = 3) or by 1.5 × 10^4^ oocysts of VEG strain by the oral route (*n* = 3), as previously described (Garcia et al., 2005). Serum samples of these animals were collected and analyzed on days 0, 7, 14, 21, and 28 post-infection.

#### Mouse Serum Samples

Sera were obtained from mice experimentally infected with *T. gondii* tissue cysts or oocysts. All experiments were carried out with 8–12 weeks-old female Balb/c mice maintained under standard conditions in the Bioterism Center and Animal Experimentation, Federal University of Uberlândia, MG, Brazil. Briefly, two groups of five animals were infected with 50 oocysts or 50 cysts of *T. gondii* (VEG strain) by oral route, as described elsewhere (Hill et al., 2011). Serum samples were collected and analyzed on days 0, 15, 30, 45, and 60 post-infection.

### Human Serum Samples

#### Serum Samples from Outbreak

A total of 78 serum samples from a toxoplasmosis outbreak was collected from individuals living in the municipalities of Ouro Preto do Oeste, Jaru and Ji-Paraná in the state of Rondônia, Brazil (Sociedade Brasileira de Parasitologia, 2011). This well-documented toxoplasmosis outbreak occurred between July and August from 2011 and the source of infection was water containing *T. gondii* oocysts, due to an accident that took place 3 weeks before, which contaminated the water resources from the cities. All infected individuals started with symptoms such as fever, prostration, muscle pain, joint and head, onset of swelling in the lymph nodes, especially in the neck region. Ocular disease was observed in some cases. All samples were seropositive for *T. gondii* infection by commercial immunoassays. The epidemiological investigations concluded that the source of infection was water contaminated by oocysts.

#### Pregnant Women

A total of 78 serum samples from pregnant women with IgM and IgG against *T. gondii* was analyzed. These samples came from the Outpatient Reference Centre for Pediatric Infectious Diseases, which is the reference service for congenital toxoplasmosis at the Outpatient Clinical Hospital, State University of Londrina, Paraná State, Brazil.

### Western Blot Analysis

#### Electrophoretic Transfer

For Western blot analysis, 2 μg of each antigen (recombinant proteins or STAg) in sample buffer (Tris-HCl 100 mM [pH 6.8]; 4% SDS; 20% glycerol, bromophenol blue 0.2%) were incubated for 5 min at 100°C and resolved by 12% SDS-PAGE. After electrophoretic separation, proteins were transferred to nitrocellulose membrane using electrotransfer system (Mini Trans-Blot^®^ Eletrophoretic Transfer Cel, Bio-Rad, Hercules, CA, USA). The nitrocellulose membranes were cut into approximately 3 mm wide strips and placed in channels for the reaction.

#### Immunoblotting of Serum Samples

Strips were blocked with PBS plus 0.05% Tween 20 (PBS-T) supplemented with 5 or 8% skimmed milk for 2 h at room temperature. After washing with PBS-T, the strips were incubated for 18 h at 4°C with serum samples from chickens, pigs or mice, as described elsewhere (Dubey, 2010). After washing with PBS-T, strips were incubated with secondary antibody specific to each species conjugated to peroxidase (Sigma) for 2 h at room temperature. The membranes were washed again with PBS-T and developed by adding 3,3′-diaminobenzidine (DAB) at 1 mg/ml in 20 mM Tris-HCl (pH 7.2) and 0.03% H_2_O_2_.

### Enzyme-linked Immunosorbent Assay

The immunoenzymatic assays were carried out to detect antibody reactivity against STAg or recombinant proteins in serum samples from chickens, pigs, mice, and humans. Briefly, high affinity polystyrene microtiter plates (Corning 3590 Laboratories, Inc., New York, NY, USA) were coated with STAg (10 mg/ml) or recombinant proteins (2 mg/ml) in carbonate-bicarbonate buffer 0.06 M (pH 9.6) for 18 h at 4°C. Plates were incubated with serum samples and subsequently with secondary antibody specific to each species conjugated to peroxidase (Sigma). The assay was revealed by adding the enzyme substrate (0.03% H_2_O_2_ and 2,2-azino-bis-3-ethyl-benzothiazoline sulfonic acid [ABTS, Sigma] in 0.07 M citrate phosphate buffer, pH 4.2). The cut off of the reaction was calculated as the mean OD of negative control sera plus 3 standard deviations. The negative control sera consisted of a pool of 10 serum samples tested negative for *T. gondii* infection. Antibody titers were expressed as ELISA index (EI), according to the following formula: EI = OD sample/OD cut off, as previously described (Silva et al., 2002). Samples with EI values ≥ 1.2 were considered positive.

### Statistics

Statistical analyses were performed using the GraphPad Prism v. 5.0 (GraphPad Software, San Diego, CA, USA). Antibody levels were compared between the recombinant proteins and STAg using one-way ANOVA with Bonferroni post-test. Student’s *t*-test was used to assess differences in the antibody kinetics analyses. Positivity rates for antigen preparations were analyzed by Fisher exact test. Values of *P* < 0.05 were considered statistically significant.

## Results

### Serologic Differentiation of *T. gondii* Stages in Chicken Infections

**FIGURE 1:**
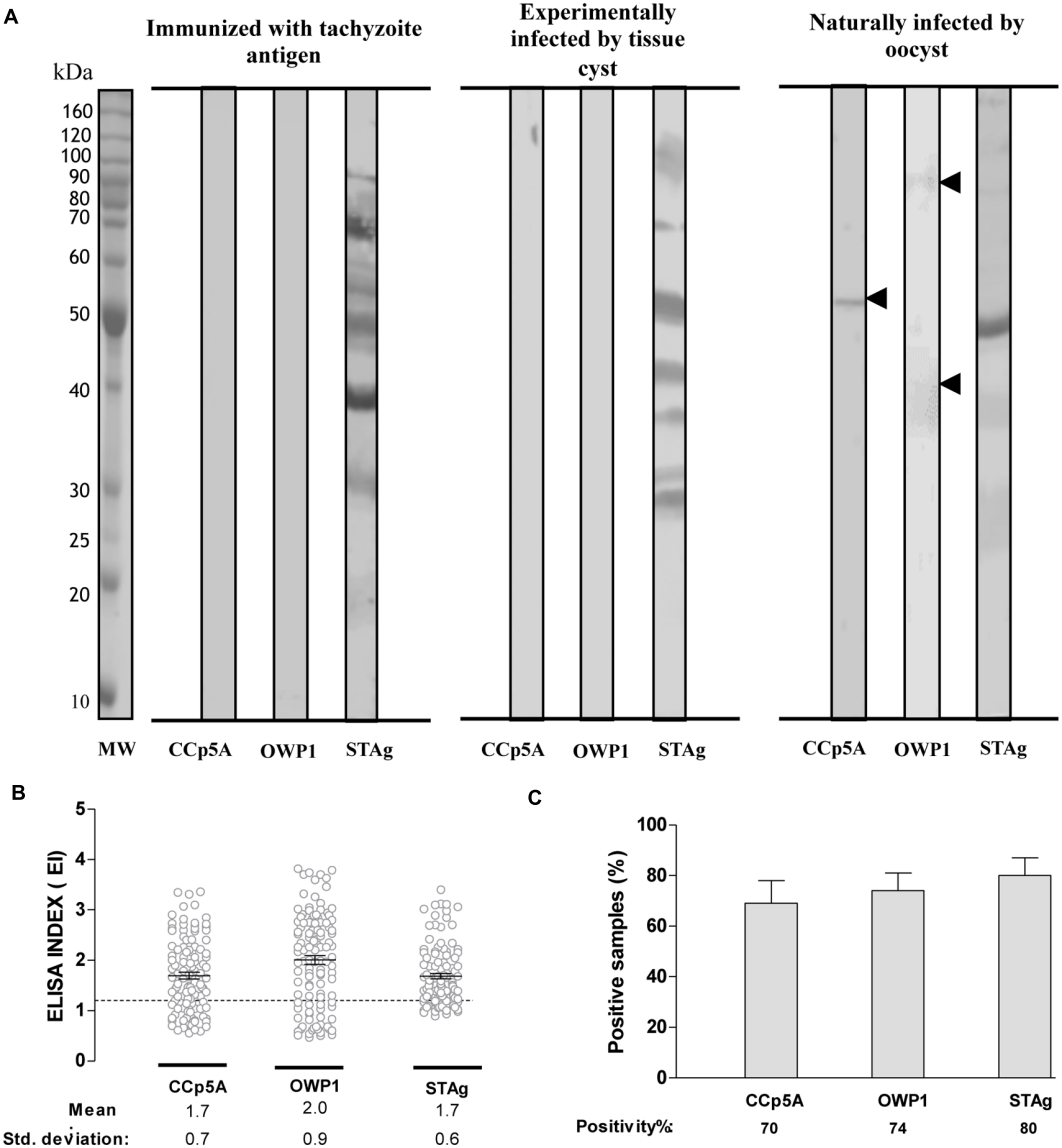
**Serologic analysis of chicken sera using oocyst/sporozoite-specific proteins of *Toxoplasma gondii*. (A)** Two recombinant proteins from *T. gondii* oocyst/sporozoite (CCp5A and OWP1) and whole tachyzoite soluble antigen (STAg) were tested by Western blot using serum samples from chickens immunized with STAg (left panel), experimentally infected with *T. gondii* tissue cysts (central panel) or naturally infected by oocysts (right panel). The arrowheads indicate the detected protein bands and the molecular weight markers (MW) are represented in kDa at the left side of left panel. Levels of IgY antibodies **(B)** and positivity **(C)** were evaluated using ELISA in serum samples from free range chickens collected in suburban areas of Uberlândia, MG, Brazil (*n* = 113). The results were expressed as ELISA index (EI) and values higher than 1.2 were considered positive. The dashed line indicates the cut off of the reactions. Significant differences among IgY levels against recombinant proteins and STAg were determined by one-way ANOVA, while the differences among positivity rates were evaluated using Fisher’s exact test.

In order to identify a *T. gondii* protein potentially able to differentiate infections driven by distinct parasite infective stages, it was analyzed serum samples from chickens by Western blot analysis on the selected *T. gondii* proteins. It was included recombinant polypeptides belonging to distinct parasite proteins whose expression suggested an oocyst/sporozoite-specific profile, as determined by proteomic analyses reported in the ToxoDB database. Chicken sera were assessed against the recombinant polypeptides in comparison with STAg. When serum samples from different group of chickens were tested against the recombinant antigens, a differential reactivity was observed for CCp5A and OWP1 polypeptides (**Figure 1A**). Both proteins were recognized exclusively by sera from naturally infected chickens, with the CCp5A protein fragment showing the highest reactivity among all analyzed recombinant proteins.

To confirm whether the recombinant fragments from CCp5A and OWP1 could work as serological markers of infection via oocysts, it was performed an ELISA against the recombinant proteins in comparison with STAg, using 113 individual sera from free-range chickens. The levels of IgY antibodies were expressed by ELISA index (EI) for each antigen. The STAg reactivity showed an EI mean of 1.7, as compared to CCp5A and OWP1, which displayed EI means of 1.7 and 2.0, respectively (**Figure 1B**). The positivity rates were 80% for STAg, 70% for CCp5A, and 74% for OWP1 (**Figure 1C**). Based on these results, CCp5A and OWP1 showed characteristics of oocyst markers for chicken infection.

### Serologic Differentiation of *T. gondii* Stages in Pig Infections

**FIGURE 2:**
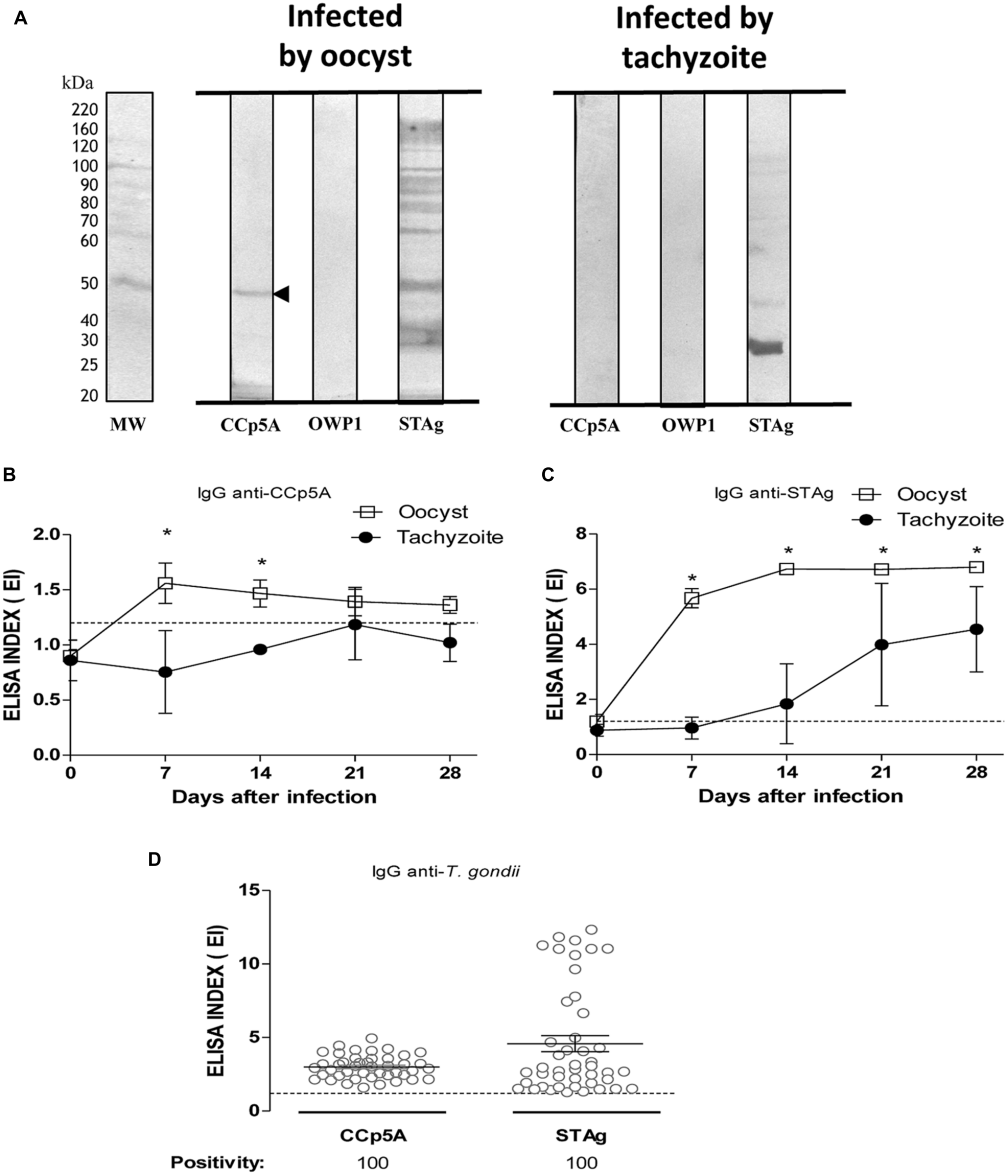
**Detection of anti-*Toxoplasma* antibodies in pig sera using the oocyst/sporozoite-specific protein CCp5A. (A)** The recombinant proteins CCp5A and OWP and STAg were tested by Western blot using serum samples from pigs experimentally infected with oocysts or tachyzoites. The arrowhead indicates the detected protein band and the molecular weight markers (MW) are represented in kDa at the left side of left panel. Kinetics of IgG production to *T. gondii* determined by ELISA using recombinant CCp5A **(B)** or STAg **(C)** in sera from pigs experimentally infected with oocysts (blank squares) or tachyzoites (black circles). **(D)** Levels of IgG antibodies and positivity rates were evaluated using ELISA in serum samples from free range pigs created in Piracaíba farm in Araguari, MG, Brazil. The results were expressed as ELISA index (EI) and values higher than 1.2 were considered positive. The dashed line indicates the cut off of the reactions. Significant differences between sources of infection were determined by the Student’s *t*-test for each time point or Fisher’s exact test to compare positivity rates. ^∗^*P* < 0.05.

To distinguish sources of *T. gondii* infection in pigs, it was assessed the recombinant proteins analyzed for chicken serum samples. As shown in **Figure 2A**, CCp5A showed exclusive reactivity for serum samples from pigs infected by oocysts, whereas OWP1 showed no reactivity for all serum samples analyzed. As expected, the STAg antigen presented reactivity for all serum samples, no matter the source of infection. As only recombinant CCp5A was able to differentiate the infective stages in pigs, it was used this antigen to evaluate its performance in the kinetics of IgG production in pigs experimentally infected by either oocysts or tachyzoites. Serum samples were collected on days 0, 7, 14, and 28 post-infection and analyzed by ELISA. As shown in **Figure 2B**, the kinetics of IgG to CCp5A in serum samples from pigs infected with oocysts showed increasing levels until day 7 post-infection (EI = 1.6). After this time point, it was observed a slight decline until day 28 post-infection (EI = 1.3). In contrast, when analyzing the kinetics of IgG to CCp5A in serum samples from tachyzoite-infected pigs, EI mean values were below the cut off (EI = 1.2) for all time points analyzed (**Figure 2C**). Also, significant differences between the sources of infection were observed on day 7 and 14 post-infection, with detection of higher levels in serum samples from pigs infected with oocysts, in comparison with serum samples from pigs infected with tachyzoites. The early detection of anti-CCp5A IgG in pigs infected with oocysts suggested that this recombinant polypeptide might be considered a serological marker of recent oocyst-driven *Toxoplasma* infection in pigs. The production of IgG against STAg in serum samples from oocyst-infected pigs raised at day 7 post-infection and increased at all time points (**Figure 2C**), showing highest levels on day 28 post-infection (EI = 6.8). Similarly, the kinetics assay for IgG to STAg in serum samples from pigs experimentally infected with tachyzoites increased over time with highest levels on day 28 post-infection (EI = 4.5; **Figure 2C**). Unlike oocyst-driven infection, positive EI values were observed only from day 14 onward. Significant differences between oocyst and tachyzoite as sources of infection were observed for all time points analyzed, with higher levels for serum samples infected by oocysts (*P* < 0.0001).

In order to assess the diagnostic value of the CCp5A recombinant polypeptide in a population of naturally infected pigs, serum samples from animals raised extensively (*n* = 44) were tested against CCp5A and STAg by ELISA. As shown in **Figure 2D**, 100% of positivity was observed for both antigenic preparations and no significant difference was observed in the levels of IgG antibodies between CCp5A and STAg. The seropositivity against CCp5A strongly suggests the oocysts as the probable source of infection in naturally infected pigs.

### Serologic Differentiation of *T. gondii* Stages Involved in Mouse Infections

**FIGURE 3:**
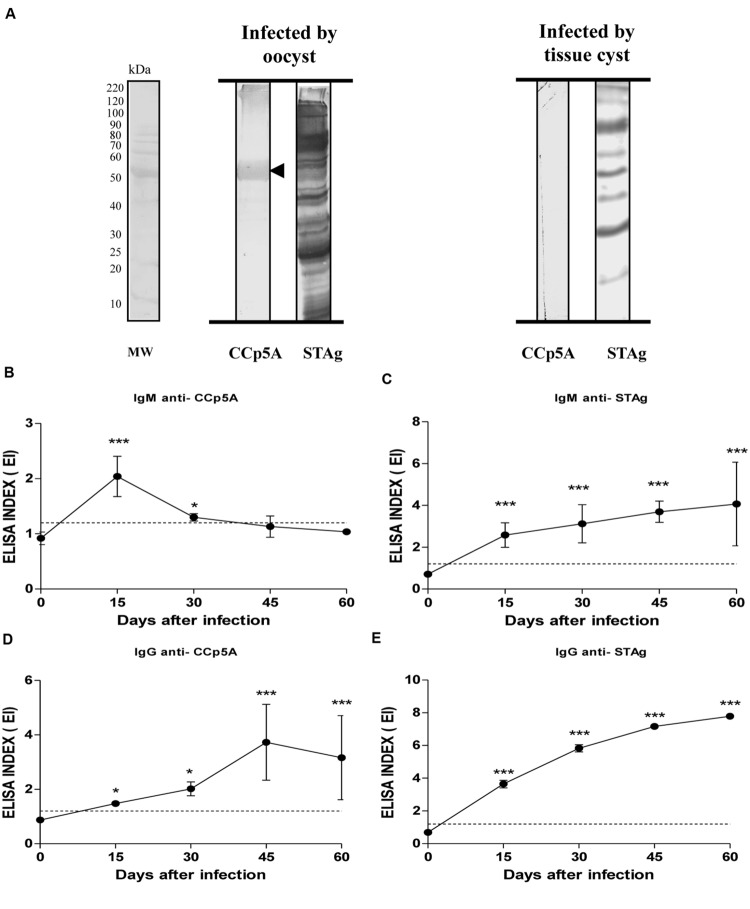
**Detection of anti-*Toxoplasma* antibodies in mouse sera using the oocyst/sporozoite-specific protein CCp5A. (A)** The CCp5A recombinant protein and STAg were tested by Western blot using serum samples from mice experimentally infected with *T. gondii* oocysts or tissue cysts. The arrowhead indicated the detected protein bands and the molecular weight markers (MW) are represented in kDa at the left side of left panel. Kinetics of IgM **(B,C)** or IgG **(D,E)** production to recombinant CCp5A **(B,D)** or STAg **(C,E)** were determined by ELISA in sera from mice experimentally infected with oocysts at different time points after infection. The results were expressed as ELISA index (EI) and values higher than 1.2 were considered positive. Dashed line indicates the cut off of the reaction. Significant differences between day 0 and other time points were determined by paired Student’s *t*-test in each time point. ^∗^*P* < 0.05; ^∗∗∗^*P* < 0.0001.

In order to assess the stage-specificity of the humoral response directed against CCp5A in a more controlled animal model, Balb/c mice were infected by oocysts or tissue cysts. As shown in **Figure 3A**, Western blot analysis revealed that CCp5A is exclusively recognized by serum samples from oocyst-infected mice, whereas STAg showed reactivity to serum samples from both oocyst- and cyst-infected mice. In agreement with the findings observed in pigs, these results confirm the ability of CCp5A to differentiate *T. gondii* stages responsible for the infection. Aiming to further evaluate the serological characteristics of CCp5A in comparison with STAg, kinetics assays for both IgM and IgG isotypes were carried out, using serum samples from mice infected by oocysts. As shown in **Figures 3B,C**, the kinetics of IgM synthesis toward CCp5A and STAg presented different profiles. Interestingly, the kinetics of IgM for CCp5A showed a peak at day 15 post-infection (EI = 2.0), followed by a marked drop of the antibody titer reaching values below the cut off at day 45 post-infection (EI = 1.1). Significant differences between day 0 and day15 (*P* < 0.0001) and day 0 and day 30 (*P* < 0.05) were observed. In contrast, IgM antibodies to STAg was found to increase over time, and the highest levels were reached at day 60 post-infection. Significant differences between day 0 and all other post-infection time points were observed (*P* < 0.0001). The kinetics of anti-CCp5A IgG production showed that the antibody titer increased over time, reaching a peak on day 45 (EI = 3.7). From day 45 post-infection, a slight decrease was observed until day 60 post-infection (EI = 3.1; **Figure 3D**). The kinetics of anti-STAg IgG showed that this isotype level increased over time, reaching a peak at day 60 post-infection (EI = 7.8; **Figure 3E**). Significant differences between day 0 and other time points were observed (*P* < 0.05; *P* < 0.0001). The kinetics of IgM production against CCp5A points out to this protein as a parasite component associated with an early stage of infection in oocyst-infected mice.

### Evaluation of CCp5A as a Serologic Marker in Human Sera

**FIGURE 4:**
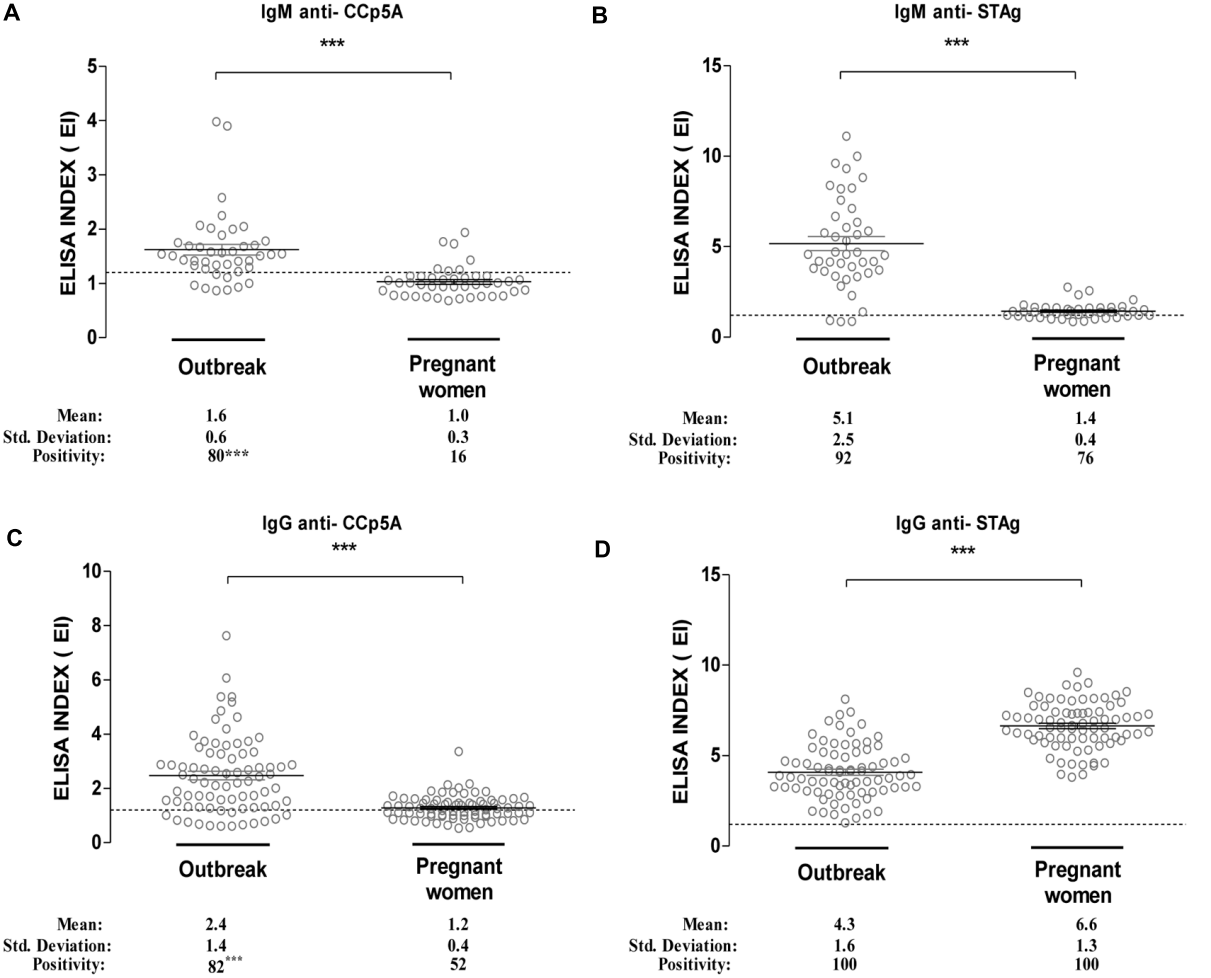
**Reactivity of recombinant CCp5A with human sera from a toxoplasmosis outbreak.** Recombinant CCp5A was used to screen human sera from patients involved in a waterborne toxoplasmosis outbreak or from pregnant women with anti-*T. gondii* IgG antibodies. The levels of IgM **(A,B)** of IgG **(C,D)** to recombinant CCp5A **(A,C)** or STAg **(B,D)** were evaluated by ELISA. The results were expressed as ELISA index (EI) and values higher than 1.2 were considered positive. Dashed line indicates the cut off of the reactions. Significant differences among serum samples levels were determined by Student’s *t*-test. ^∗∗∗^*P* < 0.0001.

As the CCp5A polypeptide resulted a stage-specific serological marker of infection in the animal species, the next step was to evaluate its performance with two different panels of human serum samples, in comparison with STAg. As shown in **Figure 4A**, the levels of IgM antibodies against CCp5A polypeptide were higher in serum samples from patients involved in toxoplasmosis outbreak (EI = 1.6), when compared with sera from pregnant women (EI = 1.0; *P* < 0.0001). Interesting, the IgM positivity rate of serum samples from the toxoplasmosis outbreak (80%) was markedly higher, when compared with serum samples from pregnant women (16%; *P* < 0.0001). We also evaluated the IgM levels and positivity rates to STAg. As shown in **Figure 4B**, the levels of IgM were significantly higher in serum samples from the outbreak (EI = 4.6), when compared with sera from pregnant women (EI = 1.4; *P* < 0.0001). However, it was observed no significant difference between the positivity rates of sera from the outbreak (92%) and pregnant women (76%). Next, it was evaluated the IgG levels and reactivity to the CCp5A recombinant fragment (**Figure 4C**). As observed for IgM, the IgG antibody levels were higher in serum samples from the outbreak (EI = 2.4), when compared with serum samples from pregnant women (EI = 1.2). Significant difference between the positivity rate of outbreak (82%) and pregnant women (52%) serum samples was observed (*P* < 0.001). In contrast, the analysis of IgG antibodies to STAg demonstrates higher levels of antibodies in sera from pregnant women (EI = 6.6), when compared with serum from the *Toxoplasma* outbreak (EI = 4.4), as shown in **Figure 4D** (*P* < 0.0001). The positivity rates for both groups of sera were 100%. Overall, these results suggest that CCp5A is able to detect both IgM and IgG antibodies in human sera from patients who have been infected by *T. gondii* oocysts. Additionally, it was observed that in serum samples from pregnant women with serological evidence of recent exposure to the parasite, anti-CCp5A IgM antibodies were detected in 80% of samples, indicating a high prevalence of oocyst-driven infection.

## Discussion

Toxoplasmosis has emerged as a waterborne disease of significant medical importance, considering its probability to cause outbreaks, with serious consequences even for immunocompetent people, who may develop severe ocular disease (Aramini et al., 1998, 1999; Burnett et al., 1998; Dubey, 2004). In these situations, oocysts shed by feces from felids, either from urban or rural areas, can contaminate water reservoirs and infect humans and domestic animals (Aramini et al., 1999; de Moura et al., 2006; Schluter et al., 2014). The clinical importance of toxoplasmosis and the possibility of causing outbreaks worldwide has led to its classification as Category B priority agent by The National Institute of Allergy and Infectious Diseases (NIAID; Kim and Weiss, 2008).

Previous studies have shown that, in intermediate hosts, the infection by oocysts is considered clinically more severe when compared to infections caused by cysts (Dubey, 2004). These findings are reinforced by the fact that many cases of human infections caused by oocysts exhibit clinical symptoms, even in immunocompetent individuals (Montoya and Liesenfeld, 2004; Innes, 2010). In addition to these relevant outcomes, oocysts are able to contaminate the environment as shown by serologic surveys conducted on herbivorous animals and vegetarian people (Rawal, 1959; Dubey, 2010; Ahmad and Qayyum, 2014). Taking into account the central role of oocysts in the *T. gondii* transmission, the identification of proteins that are expressed exclusively by oocysts/sporozoites may help to reveal parasite targets for the development of novel stage-specific serologic assays (Fritz et al., 2012b; Possenti et al., 2013).

The search for specific proteins from *T. gondii* oocysts/sporozoites has been investigated since the 80s (Kasper and Ware, 1985; Kasper, 1989), but only recently oocyst/sporozoite-specific proteins have been characterized with potential use in vaccine design and diagnosis (Radke et al., 2004; Possenti et al., 2010; Boyer et al., 2011; Hill et al., 2011; Doskaya et al., 2014). In the present study, it was investigated the serologic application of *T. gondii* polypeptides specific from oocysts/sporozoites by using reference serum samples from animals (chickens, pigs, and mouse) and humans, in order to determine which parasite stage was involved in the infection. First, the entire panel of recombinant proteins was tested against sera from chickens, naturally infected by oocysts, experimentally infected with tissue cysts, or experimentally immunized with tachyzoite antigen. Based on the results obtained by Western blot and ELISA, the proteins CCp5A and OWP1 were selected to be further analyzed by ELISA against 113 serum samples from naturally infected chickens. These proteins showed ability to detect antibodies preferentially in sera from animals infected by *T. gondii* oocysts, reinforcing the results obtained by Western blot. High positivity rates of naturally infected chicken sera were found for CCp5A (70%), and OWP1 (74%), demonstrating the high level of environmental contamination with oocysts in the region. To our knowledge, this is the first work using sera from hens in order to differentiate infective stages of *T. gondii*. Moreover, the majority of studies performed to analyze antibody response in chickens against *T. gondii* used total antigens of the parasite (Zhu et al., 2008; Barakat et al., 2012; Zhao et al., 2012). It was described in the literature just a single work evaluating the use of recombinant proteins from *T. gondii* oocysts against serum samples from chickens (Hotop et al., 2014). However, the authors only evaluated the correlation between the immune response toward those recombinant proteins and the dose of oocysts experimentally inoculated in chickens. In contrast, our study focused on the identification of the parasite stage responsible for infection, indicating that recombinant CCp5A and OWP1 showed predominant reactivity with sera from chickens infected by oocysts. Next, we probed the recombinant proteins CCp5A and OWP1 with sera from pigs in order to assess the ability to differentiate the infective forms of *T. gondii* in a mammalian model that is known to be a significant source of *Toxoplasma* infections in humans (Dubey, 2009; Schluter et al., 2014). Only CCp5A showed reactivity with serum samples from experimentally infected pigs. The lack of reactivity of OWP1 with serum samples from pigs may be due to differences in the immune system of mammals and birds (Sharma and Tizard, 1984; Butler et al., 2009). Thus, our data indicate that CCp5A may be used to identify sera from chickens and pigs infected with oocysts.

When the kinetics of IgG in serum samples from pigs infected with oocysts was analyzed, the CCp5A protein showed higher reactivity to this antibody isotype at day 7 post-infection. From this early phase onward, the kinetics assays showed a tendency to decrease over the time. On the other hand, the kinetics assays for IgG detection to CCp5A in serum samples from animals infected with tachyzoites showed no reactivity, as all EI values were below the cut off at all time points. These results strengthen the specificity of CCp5A to detect anti-*Toxoplasma* antibodies in pigs infected with oocysts and indicate that it could be considered a molecular marker for acute phase of infection. In contrast, the IgG kinetics to STAg increased over time for sera from animals experimentally infected by oocysts or tachyzoites. Taken into account that these differences in the kinetics of IgG production are likely due to the type of infective stage, the early detection of IgG against CCp5A observed in our study might be due to the expression of this protein only by sporozoites, whose stage-specific antigens are exposed to the host immune system exclusively at the beginning of the infection (Dubey et al., 1998). Accordingly, CCp5A, which contains a LCCL domain (Trexler et al., 2000), a common feature of various secreted proteins among apicomplexan parasites (Tosini et al., 2006), including *T. gondii*, is expressed only in the oocyst stage (Fritz et al., 2012a). It was described in the literature that a recombinant protein from *T. gondii* sporozoites (TgERP) is able to differentiate infection by cysts or oocysts in serum samples from pigs by Western blot (Hill et al., 2011). It was observed that this antigen preparation was able to selectively detect the presence of specific antibodies to TgERP in pigs infected with oocysts until 6–8 months after infection In our work, however, the highest level of anti-CCp5A IgG antibodies in oocyst-infected pigs was detected at day 7 post-infection, followed by a gradual decrease until day 28. These observations confirm the importance of the antigenic preparations to evaluate antibody kinetics, particularly whether they are prepared from sporozoite, bradyzoite, or tachyzoite parasites. In addition, our data showed 100% of positivity to CCp5A and STAg in samples from pigs raised extensively, resulting in naturally infected animals and indicating that the causative agent was the oocyst and suggesting a high level of environmental contamination by oocysts on the farm, where they had been raised. Similar results were observed using the TgERP protein in pig samples from slaughterhouses in Chile (80% of positivity; Munoz-Zanzi et al., 2012). These observations show the impact of the environmental contamination by oocysts in infected animals, which are used as food resources for humans, making clear that the identification of the infective form responsible for the infection is important.

The reactivity of CCp5A protein assessed in the mouse model was confirmed with sera from animals infected with oocysts, as a strong staining by Western blot was observed in the present study. The kinetics approaches for IgM and IgG antibodies to STAg in mice infected with oocysts revealed increasing levels over time. In contrast, the kinetics of IgM and IgG antibodies to CCp5A presented different profiles, with IgG antibodies to CCp5A reaching the highest level at 45 days after infection. As noticed for the kinetics of IgG antibodies in pigs infected with oocysts, the kinetics of anti-CCp5A IgM production in mice showed an early peak of reactivity, at day 15 post-infection. In agreement with our results, a previous study assessing the kinetics of mouse IgM antibodies against a recombinant protein from *T. gondii* sporozoites (SporoSAG), showed a peak of reactivity from 10 to 15 days post-infection in mice infected with oocysts (Doskaya et al., 2014), reinforcing the hypothesis that the use of proteins expressed exclusively by oocysts/sporozoites is critical to identify the early phase of oocyst-driven *T. gondii* infection.

A major obstacle to carry out procedures against toxoplasmosis in humans is the lack of information concerning the infective form of *T. gondii* responsible for the infection in a given population. In order to overcome this difficulty, we have selected the CCp5A protein by screening panels of serum samples from various intermediate hosts, including a panel of human sera. This latter came from patients involved in a toxoplasmosis outbreak, which epidemiological investigations revealed that the affected individuals were likely infected by ingestion of oocysts in water. Also, this panel included serum samples from pregnant women showing positivity for IgM and IgG antibodies to *T. gondii*, but whose source of infection was unknown. When these serum samples were assessed for IgM antibodies against STAg, no significant differences were observed in the percentages of positivity between both groups (92% for outbreak-related sera and 76% for serum samples from pregnant women), although the reactivity levels were higher in serum samples from the outbreak. For IgG antibodies, the percentage of positivity was 100% for both groups of infected individuals, being these results in accordance with those observed for animal sera (chickens, pigs, and mouse), in which the STAg could not differentiate the infective forms of the parasite. However, when we screened for IgM and IgG directed to CCp5A, it was observed higher antibody levels and positivity rates in serum samples from the outbreak, as compared with samples from pregnant women. When analyzing the IgM antibodies, it was observed that 80% of the serum samples showed reactivity to CCp5A, whereas only 16% of sera from pregnant women were positive. These results suggest that the majority of the patients from the outbreak, who were presenting clinical manifestations of acute infection, were actually infected by oocysts, supporting the hypothesis that CCp5A shows characteristics of a molecular marker for recent infection by *T. gondii*. When it was compared the percentage of IgM positivity between STAg (76%) and CCp5A (16%) in serum samples from pregnant women, a significant difference could be confirmed. This difference may be due to the fact that STAg presents a mixture of various proteins, which may lead to detection of IgM antibodies beyond the acute phase. On the other hand, the lower detection of anti-CCp5A IgM observed in serum samples from pregnant women infected by an unknown parasite stage, can be explained by the fact that a single protein was employed. The use of a single, well-defined antigen like CCp5A can reduce the frequency of false positive results, which are frequently observed in conventional immunoassays. For IgG analysis, 82% of outbreak serum samples were positive for CCp5A, while 52% of pregnant women sera were positive. These results indicate that the majority of the outbreak serum samples came from patients that were actually infected by oocysts, while 18% of serum samples came from patients infected by other parasite stages. The serologic analysis of sera from pregnant women showed that 52% of samples reacted with CCp5A. These results indicate that pregnant women in this population might be infected by oocysts or tissue cysts with equal probability, showing the importance to prevent the infection during pregnancy not only by avoiding the ingestion of undercooked meat, but also preventing contact with oocysts present the environment.

In a previous study using the recombinant protein from sporozoite (TgERP) against human serum samples infected with oocysts, Hill et al. (2011) evaluated 17 serum samples infected by oocysts, being six samples from laboratory employees that were accidentally exposed to oocysts and 11 serum samples from outbreak of toxoplasmosis. All six infected individuals presented IgM and IgG antibodies to TgERP and 9 out of 11 (82%) individuals infected with oocysts had detectable antibodies to TgERP. These results are in agreement with the present study, although we have used a higher number of serum samples from outbreak to observe our results.

In summary, we have demonstrated that CCp5A is a new serologic tool capable to identify oocysts/sporozoites as the causative agent for *T. gondii* infection. This parasite sporozoite-specific protein can be considered a new molecular marker to be used in diagnosis and epidemiological studies in order to identify the source of infection for animals and humans. The identification of the infective stage of *T. gondii* responsible for the infection can help to design effective strategies to minimize parasite transmission and to prevent severe complications, mainly in immunocompromised people and pregnant women. Furthermore, these findings contribute to identify the source and parasite stage responsible for *T. gondii* infection in cases of toxoplasmosis outbreaks. Future studies should be done to evaluate the immunogenic ability of the CCp5A protein in vaccination protocols.

## Conflict of Interest Statement

The authors declare that the research was conducted in the absence of any commercial or financial relationships that could be construed as a potential conflict of interest.
